# Prevalence of *Leptospira* in murine in China: A systematic review and meta-analysis

**DOI:** 10.3389/fvets.2022.944282

**Published:** 2022-09-29

**Authors:** Jian-Ming Li, Lian-Min Li, Jun-Feng Shi, Ting Li, Qi Wang, Qing-Xia Ma, Wei Zheng, Hai-Feng Feng, Fei Liu, Rui Du

**Affiliations:** ^1^College of Animal Science and Technology, Jilin Agricultural University, Changchun, China; ^2^College of Chinese Medicine Materials, Jilin Agricultural University, Changchun, China; ^3^Laboratory of Production and Product Application of Sika Deer of Jilin, Jilin Agricultural University, Changchun, China; ^4^Animal Health Supervision Institute of Jilin Province, Changchun, China; ^5^Key Laboratory of Animal Production, Product Quality and Security, Ministry of Education, Jilin Agricultural University, Changchun, China

**Keywords:** *Leptospira*, epidemiology, murine, Mice, rat, China, meta-analysis

## Abstract

Leptospirosis is an acute infectious disease caused by pathogenic bacteria from the genus *Leptospira*. The disease is widely distributed throughout China, causing harm to human and animal health. Murine may naturally carry a variety of pathogenic *Leptospira*, thus being important sources of infection by humans and livestock. The aim of this study was to assess and analyse the prevalence of *Leptospira* and its risk factors in murine. We collected 46 publications published between inception and 2022 through China Knowledge Network (CNKI), VIP Chinese Journal Database, Wanfang Database, PubMed, and ScienceDirect. In these studies, a total of 54,051 murine in 5 regions of China were investigated, and the prevalence of leptospirosis ranged from 1.11 to 35.29%. The prevalence of murine leptospirosis in south China was the highest, at 20.13%, and the lowest in northeast China, at 1.11% (*P* < 0.05). The prevalence of leptospirosis in male murine was 21.38%, which was significantly higher than that in females (17.07%; *P* < 0.05). Results according to detection method subgroup showed that the prevalence from serological testing was 15.94%, which was significantly higher than that of etiology and molecular biology methods (*P* < 0.01). In the sample subgroup, the positive rate of serum samples was 15.30%, which was significantly higher than that of tissue samples, at 7.97%. In addition, the influence of different geographical factors on prevalence was analyzed, indicating that the Yangtze River Basin was a high-incidence area for leptospirosis. The study showed that *Leptospira* were ubiquitous throughout the country, and factors such as environment, temperature and landform affect the murine distribution and their bacteria carrying rate. We suggest strengthening the continuous monitoring of leptospirosis and taking effective and comprehensive measures such as reducing water contact, vaccinating in high-incidence seasons, and avoiding human contamination caused by water pollution and contact with infected murine.

## Introduction

Leptospirosis is a global zoonosis caused by pathogenic bacteria from the genus *Leptospira*. The infection occurs especially in tropical and subtropical regions and can cause symptomatic disease in humans and many species of animals (1, 2). *Leptospira* can be divided into three evolutionary lineages (pathogenic, intermediate, and saprophytic), with more than 300 serovars (3, 4). It is a highly heterogeneous bacterial genus, and there are great differences in the prevailing species and distribution in different places. However, because of a lack of cross-immunity among various types, preventing and controlling the resulting disease is extremely difficult (5).

Murine are important disease reservoirs, with about 90% species worldwide carrying more than 200 different pathogenic microorganisms. As many as 57 types of these microorganisms are pathogenic to humans, causing 31 viral diseases, 14 bacterial diseases, 5 rickettsial diseases, and 7 parasitic diseases (6, 7). In many natural foci, the most active diseases are murine-borne infectious diseases. Previous outbreaks of murine-borne diseases have brought destructive disasters to human society (8). Since 2001 alone, there have been 14 outbreaks of infectious diseases in the world (9). In China, murine have been confirmed to carry the rabies virus (10), Japanese encephalitis virus (11), tick-borne rickettsia (12), leptospirosis and other zoonotic pathogens. In recent years especially, a variety of subtypes and new pathogens causing murine-borne infectious diseases have been discovered, making the prevention and control of murine-borne diseases increasingly necessary (13).

Murine can naturally carry a variety of pathogenic *Leptospira*, and they are the most important source of infection of leptospirosis in humans and livestock. *Leptospira* excreted through urine can survive in contaminated water or soil for several months. Leptospirosis in humans is mostly acquired through direct contact with infected animals or indirect contact with urine-contaminated environments (14, 15). Most infected people have subclinical or mild symptoms, but if not diagnosed and treated early may progress to a severe disease characterized by liver, kidney or lung dysfunction, or bleeding manifestations. Symptoms such as pulmonary haemorrhagic syndrome may also appear in the early stage in a few infected persons (16).

Leptospirosis is widely distributed worldwide and is more prevalent in tropical and subtropical regions. At least 200 species of animals in the world have been reported as natural carriers of pathogenic *Leptospira*, and 67 wild and domestic species in mainland China have been proven to host pathogenic *Leptospira* (17). China is one of the countries with a relatively high burden of leptospirosis, in only a few provinces and autonomous regions such as Qinghai, Xinjiang and Gansu has the disease not been found. Other regions particularly affected are Guangdong and Sichuan (17). Although the overall incidence of leptospirosis in China is currently at a low level, outbreaks still occur in some areas due to factors such as climate and changes in host animal populations (18).

No systematic analysis of the prevalence of murine *Leptospira* has been conducted in China. This study employed a systematic review and meta-analysis to analyze the prevalence of murine leptospirosis to assess potential risk factors associated with the disease. This investigation can help to understand the conditions that may favor the infection, which will have significance in guiding risk assessments to prevent epidemics of human leptospirosis.

## Methods

### Search strategy and selection criteria

We used the PRISMA reporting system to report the results of our systematic review and meta-analysis (19) and retrieved articles from the following five databases: ScienceDirect, PubMed, Chinese Web of Knowledge, the VIP Chinese journal database, and Wanfang database. All English or Chinese papers on murine leptospirosis published between database inception and January 28, 2022 were included in our research scope. The search strategy was presented in Supplementary Table S2.

The following criteria were used in the selection of studies: (1) The study must have detected the prevalence of leptospirosis in murine in China. (2) It must have included the total number of tested animals and the number of positives. (3) Articles had to be published in Chinese or English; and (4) each sample must have been from a single animal (not a pooled sample). Studies inconsistent with all the above criteria were removed. Duplicate studies, review studies and data of which full-text access was not obtained were also excluded.

### Data extraction and quality assessment

We collected the following information from the incorporated studies: first author, publication year, sampling year, geographic region, province, sample type, sex, breed, season, detection method, *Leptospira* serovars, leptospirosis prevalence and the quality of research. We further assessed the impact of geographic factors on this study, including humidity (60–70% *vs*. others), latitude (21–25 degrees *vs*. others), longitude (100–110 degrees *vs*. others), precipitation (500–1500 mm *vs*. others), altitude (0–100 m *vs*. others), average annual temperature (10–15°C *vs*. others) and topography (mountainous *vs*. others). The database was established using Microsoft Excel (version 16.32).

The quality of each study was assessed according to the grading criteria of the Recommended Assessment, Development and Evaluation (GRADE) method (20–22). If the study clearly described the detection method, sampling method and timing, and random sampling, and there were four or more potential risk factors, each item was awarded 1 point. The studies were divided into three grades: 0–1 point, 2–3 points, 4–5 points.

### Statistical analysis

Data synthesis was performed using the “meta” package in R (version 4.0.0) software (23). On the basis of previous research, we used the double-arcsine transformation method (PFT) to perform a combined calculation of rates prior to the meta-analysis [(24–26), Table 1]. We applied a random-effects model to combine total effect size and subgroup analysis to avoid high heterogeneity owing to paired analysis. Heterogeneity was predicted using *I*^*2*^ and Cochrane Q statistics (expressed as χ^2^ and *P*-values), with an *I*^*2*^ value of 25% corresponding to low heterogeneity, 50% to moderate heterogeneity and 75% to high heterogeneity. A funnel plot and Egger's test were used to assess publication bias, and trim-and-fill analysis was used to adjust publication bias. The stability of the results was verified by sensitivity analysis. Through subgroup analysis and univariate regression analysis we identified factors contributing to the heterogeneity. Survey factors include region (eastern China and other regions), sampling year (1960 to 2009 and 2010 to 2020), detection method (serological testing methods include enzyme linked immunosorbent assay, modified agglutination test, hemagglutination test, complement fixation test; etiological testing methods include isolating culture, silver impregnation; and nucleic acid detection include polymerase chain reaction), sample (serum and tissues), sex (female *vs*. male), season (spring, summer and autumn), *Leptospira* serovars (*Leptospira borgpetersenii* serovar *Ballum, Leptospira kirschneri* serovar *Pomona*, etc.), murine species (*Niviventer coninga, Rattus nitidus, Rattus norvegicus*, etc.) and study quality (high and medium).

**Table 1:** Normal distribution test and conversion of the normal distribution.

	**W**	**P**
PRAW	0.89152	0.000451
PLN	0.9567	0.08531
PLOGIT	0.96938	0.2627
PAS	0.95841	0.09942
PFT	0.95722	0.08933

W, weight; PRAW, original rate; PLN, logarithmic conversion; PLOGIT, logit transformation; PAS, arcsine transformation; PFT, double-arcsine transformation.

## Results

**Figure 1:**
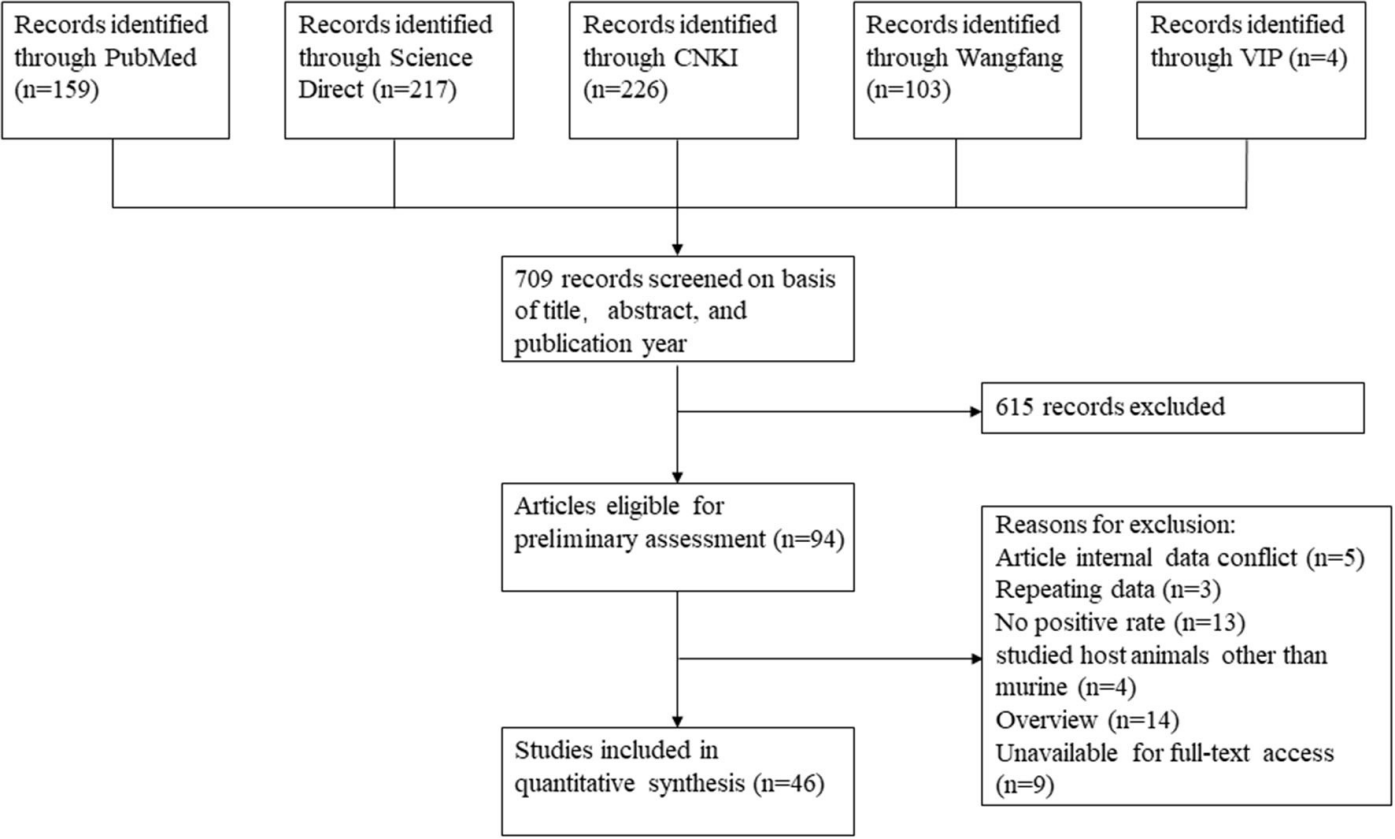
Flow chart showing the study selection process for inclusion and exclusion studies.

**Figure 2:**
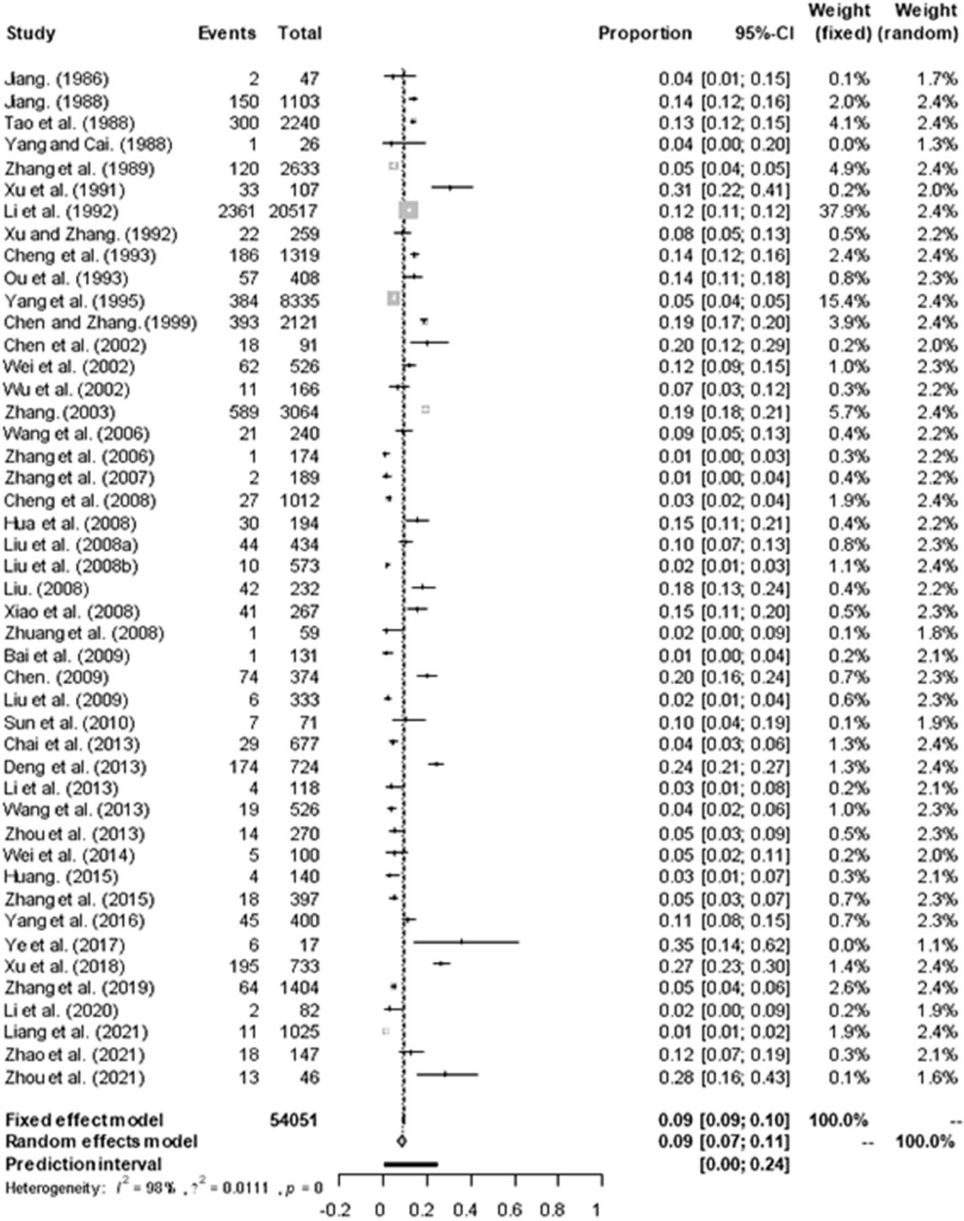
Forest plot of the prevalence of *Leptospira* in murine of China. “Study” represents the included studies; “Events” is the number of positive cases of Leptospira murine; “Total” is the total number of samples in each group; “Proportion” represents the prevalence, “CI” represents the confidence interval, “Weight” is the representative weight in the fixed and random models. The gray diamond at the bottom represents the total prevalence, the long vertical dotted line in the middle represents the meta-analysis results, and the intersection with the horizontal axis is the total OR value of 0.09, the short horizontal line represents the confidence interval of the study, the position of the short vertical line represents the OR value of each study, and the size of the short vertical line represents the weight.

**Figure 3:**
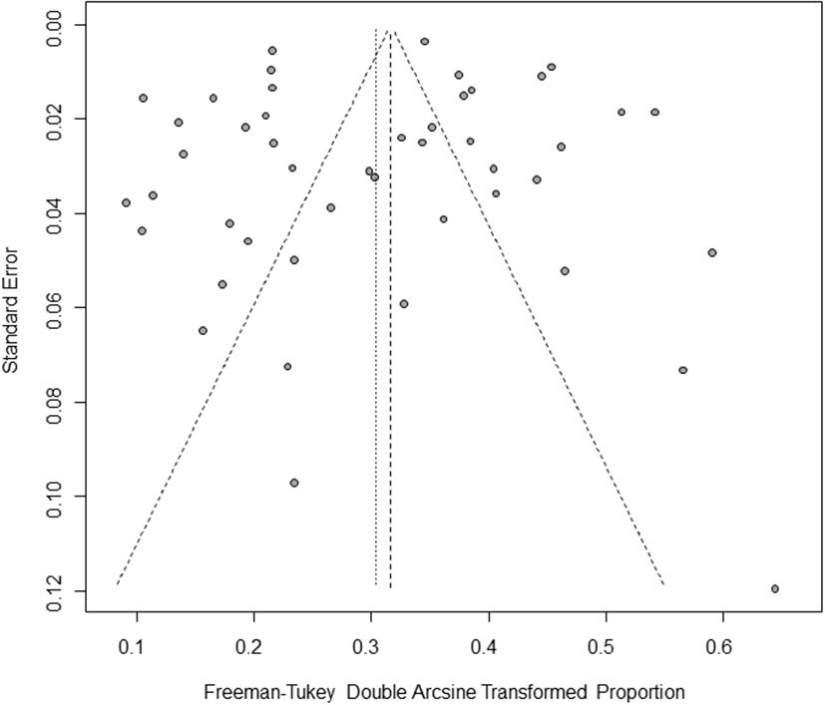
Funnel plot with pseudo 95% confidence interval limits for the examination of publication bias. The horizontal axis represents effect size, which is a measure of prevalence estimates (double arcsine transform). The vertical axis represents the transformed standard error. The gray origin represents the original study. The vertical line in the middle represents the combined effect (or main effect), and the slashed area on both sides of the vertical line represents the 95% confidence interval of the combined effect at different standard error scales. Since the slashes on both sides are not strictly 95% confidence intervals, they are called “pseudo 95% confidence limits”.

**Figure 4:**
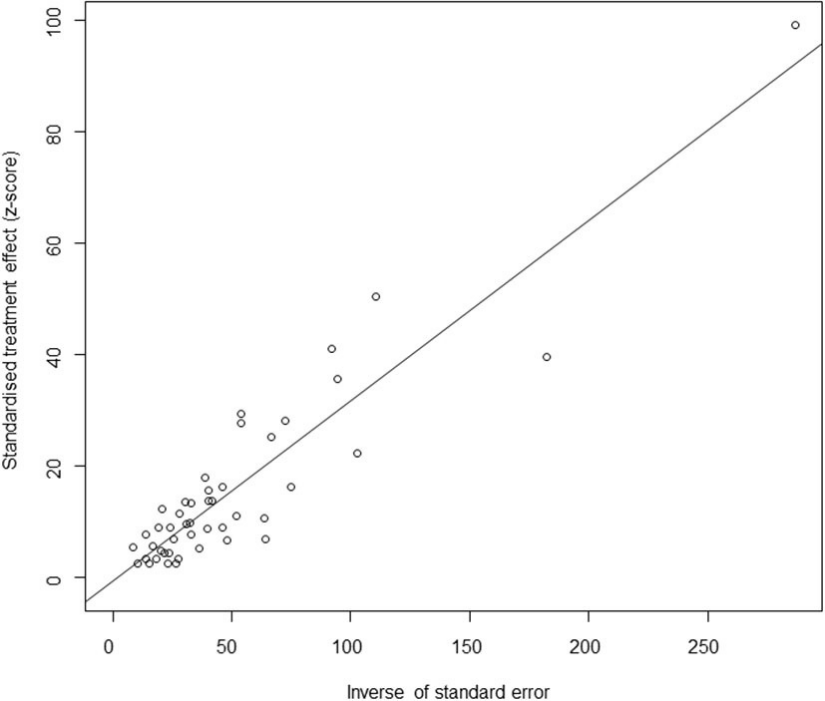
Egger's test for publication bias. Egger's test builds a simple linear regression model with the standardized effect estimator as the dependent variable and the precision of the effect estimator (the inverse of the standard error) as the independent variable. Circles represent each included study.

**Figure 5:**
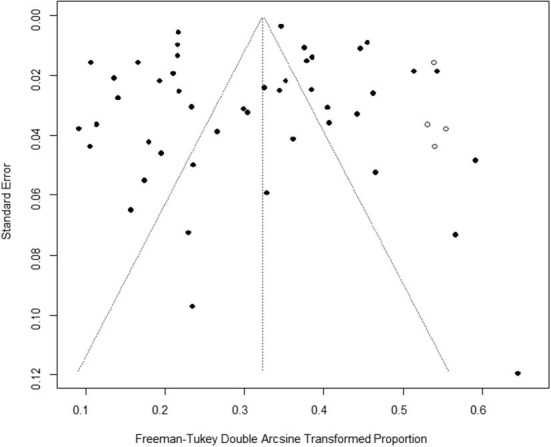
Trim-and-fill analysis. The vertical axis is the standard error, the horizontal axis is the Freeman-Tukey double Arcsine Transformed Proportion, the black dot represents the included studies, and the white dot represents the virtual studies.

**Figure 6:**
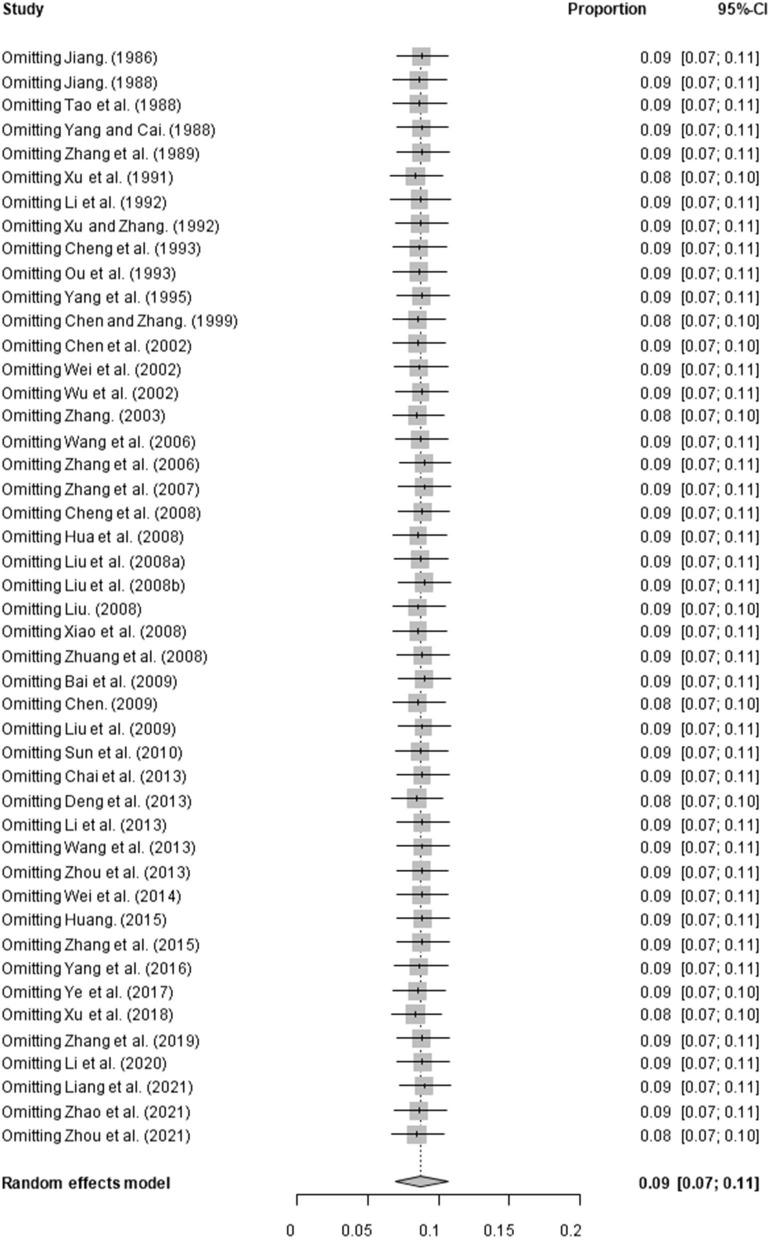
Sensitivity analysis. “Study” represents omitting study; “Proportion” represents pooled prevalence. The gray diamond at the bottom represents the total prevalence, the short horizontal line represents the confidence interval for the study. The short vertical line corresponding to each study represents the combined effect of the remaining studies after deleting the study, and the size of the gray square represents the size of the confidence interval.

Based on our search criteria, we searched five databases and performed a meta-analysis of 46 publications, including 37 high-quality papers (4 points or 5 points) and 9 medium-quality papers (2 points or 3 points) (Figure 1). The choice of a random-effects model for the meta-analysis was appropriate. The results of the forest plot showed that the study had high heterogeneity (χ^2^ = 2012.16, *I*^*2*^ = 98%, *P* = 0.00; Figure 2). We used funnel plots and Egger's test to determine heterogeneity or publication bias (Figures 3, 4) and no significant publication bias was found (t = −0.489, *P* = 0.628) (Supplementary Table S3). Trim-and-fill analysis indicated that some studies would be included, but the effect on publication bias was not significant and the findings were relatively robust (Figure 5). In conclusion, our study was free of publication bias, but other heterogeneity or minor study effect bias may have been present. In addition, we further assessed publication bias in all subgroups using funnel plots (Supplementary Figures S1–S11). Sensitivity analyses revealed that none of the studies had a significant effect on the pooled prevalence of leptospirosis; therefore, we affirmed the reliability of our meta-analysis (Figure 6).

**Figure 7:**
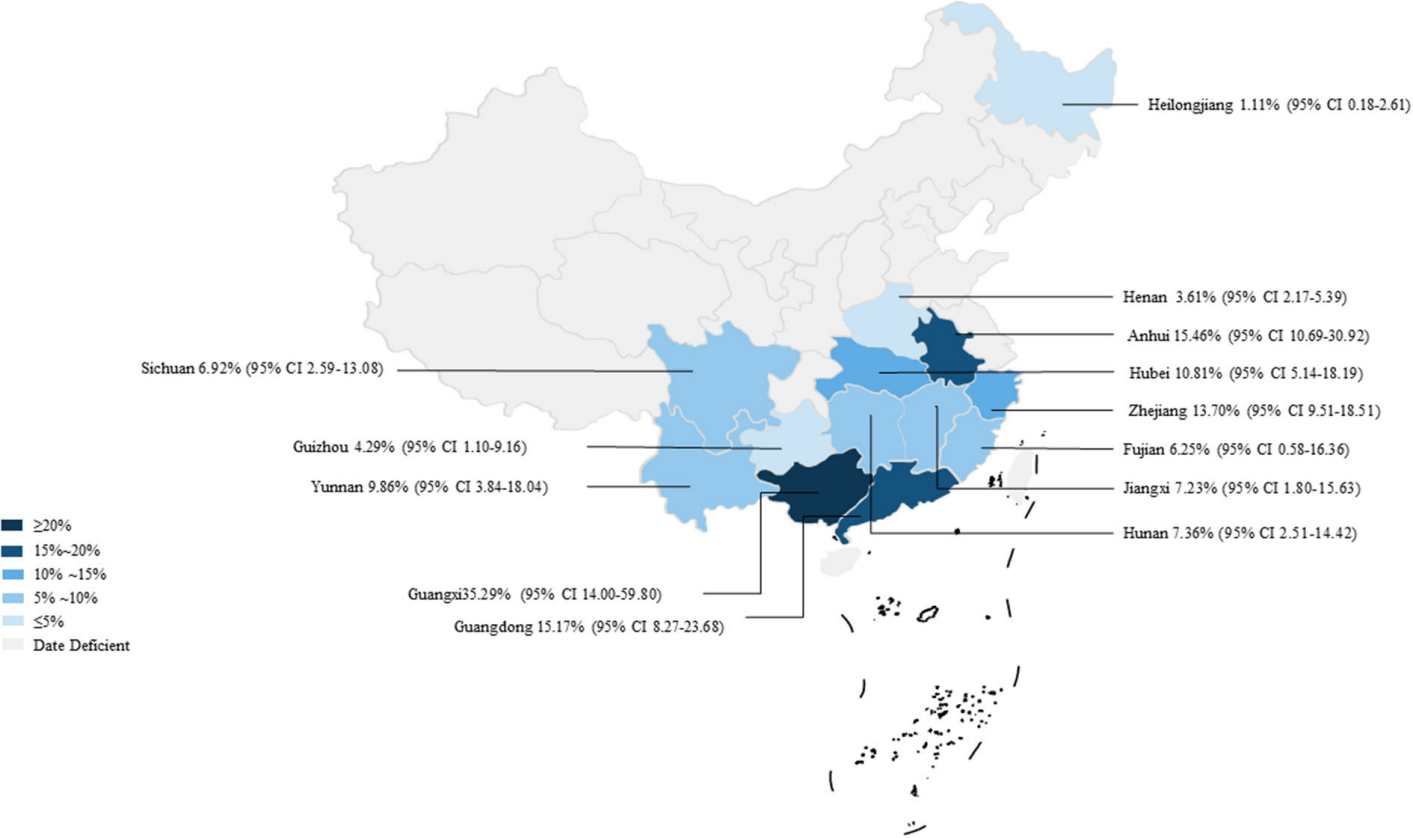
Map of *Leptospira* in murine in China. Different saturations in the HSB slider represent different infection rates.

In 46 studies, a total of 54,051 murine in 5 regions of China were investigated, with leptospirosis prevalence ranging from 1.11 to 35.29% (Table 2, Figure 7). The pooled prevalence of *Leptospira* in murine was 8.7% (95% confidence interval [CI]: 6.93–10.64%, 5617/54051; Table 4). We performed a subgroup analysis of murine species and found that the prevalence of different murine *Leptospira* species ranged from 0.00 to 29.04% (*P* < 0.05) (Table 5), and the prevalence of different *Leptospira* serovars was between 0.00 to 7.05% (Table 4). Analyzed from a geographic point of view, the prevalence of leptospirosis in murine was the highest in southern China at 20.13% (95%CI: 9.36%−33.51%, 225/1141) and the lowest in northeast China at 1.11% (95%CI: 0.18%−2.61%, 13/1107); the difference was statistically significant (*P* < 0.05).

**Table 2:** Included studies of leptospirosis in murine.

	**Province**	**Detection methods**	**Positive samples/ Total samples**	**Quality score**	**Study quality**
**Central China**					
Wang et al. (27)	Hubei	Etiological testing	21/240	4	High
Cheng et al. (28)	Hubei	Etiological testing	27/2,012	4	High
Liu et al. (29)	Hunan	Etiological testing	44/434	4	High
Liu et al. (30)	Hunan	Etiological testing	10/573	4	High
Liu (31)	Hubei	Etiological testing	42/232	4	High
Xiao et al. (32)	Hunan	Etiological testing	41/267	4	High
Chen (33)	Hubei	Etiological testing	74/374	4	High
Liu et al. (34)	Hunan	Etiological testing	6/333	4	High
Wang et al. (35)	Henan	Etiological testing	19/526	4	High
Jiang (36)	Hunan	Etiological testing	150/1,103	4	High
Cheng et al. (37)	Hubei	Etiological testing	186/1,319	3	Middle
Wu et al. (38)	Hubei	Etiological testing	11/166	3	Middle
**Eastern China**					
Jiang (39)	Jiangxi	Etiological testing/Serological testing	1/19	4	High
Xu et al. (40)	Zhejiang	Etiological testing	33/107	4	High
Xu and Zhang (41)	Zhejiang	Etiological testing/Serological testing	22/259	4	High
Chen and Zhang (42)	Zhejiang	Etiological testing	393/2,121	2	Middle
Chen et al. (43)	Zhejiang	Serological testing	18/91	3	Middle
Zhang (44)	Zhejiang	Etiological testing	589/3,064	3	Middle
Tao et al. (45)	Jiangxi	Etiological testing	300/2,240	4	High
Zhao et al. (46)	Zhejiang	Nucleic acid testing	18/147	5	High
Zhang et al. (47)	Zhejiang	Serological testing	18/397	4	High
Xu et al. (48)	Fujian	Serological testing	195/733	4	High
Zhang et al. (49)	Jiangxi	Etiological testing	64/1,404	5	High
Hua et al. (50)	Anhui	Etiological testing	30/194	4	High
Zhuang et al. (51)	Fujian	Etiological testing	1/59	3	Middle
Chai et al. (52)	Fujian	Nucleic acid testing	29/677	4	High
Zhou et al. (53)	Fujian	Nucleic acid testing	14/270	5	High
Wei et al. (54)	Zhejiang	Etiological testing	5/100	4	High
Huang (55)	Fujian	Nucleic acid testing	4/140	4	High
Yang and Cai (56)	Fujian	Serological testing	1/26	4	High
**Northeastern China**					
Li et al. (57)	Heilongjiang	UN	2/77	4	High
Liang et al. (58)	Heilongjiang	UN	11/1,025	3	Middle
**Southern China**					
Yang et al. (59)	Guangdong	Nucleic acid testing	45/400	4	High
Ye et al. (60)	Guangxi	Nucleic acid testing	6/17	5	High
Deng et al. (61)	Guangdong	Serological testing	174/724	4	High
**Southwestern China**					
Zhang et al. (62)	Guizhou	Etiological testing	1/174	4	High
Zhang et al. (63)	Sichuan	Etiological testing	2/189	5	High
Zhang et al. (64)	Sichuan	Etiological testing	120/2,633	5	High
Li et al. (65)	Guangzhou	Etiological testing	2361/20,517	3	Middle
Ou et al. (66)	Sichuan	Etiological testing	57/408	4	High
Yang et al. (67)	Guizhou	Etiological testing	384/8,335	5	High
Wei et al. (68)	Sichuan	Etiological testing	62/526	4	High
Zhou et al. (69)	Guizhou	Nucleic acid testing	13/46	5	High
Bai et al. (70)	Guizhou	Etiological testing	1/131	4	High
Sun et al. (71)	Yunnan	Nucleic acid testing	7/71	4	High
Li et al. (72)	Guizhou	Etiological testing	4/118	5	High

**Table 3:** Pooled prevalence of leptospirosis infection in murine in China.

		**No. studies**	**No. tested**	**No. positive**	**% (95% CI)**	**Heterogeneity**			**Univariate meta-regression**	
						**χ^2^**	***P*–value**	***I^2^* (%)**	***P*–value**	**Coefficient (95% CI)**
Regions^*^									0.0126	0.174 (0.038–0.311)
	Central China	12	6,579	631	8.60% (5.15–12.81)	330.09	< 0.01	96.7%		
	Eastern China	18	12,076	1,736	10.16% (6.96–13.87)	553.96	< 0.01	96.9%		
	Northeastern China	2	1,107	13	1.11% (0.18–2.61)	1.39	0.24	28.2%		
	Southern China	3	1,141	225	20.13% (9.36–33.51)	32.08	<0.01	93.8%		
	Southwestern China	11	33,148	3,012	6.28% (3.73–9.40)	594.64	<0.01	98.3%		
Provinces^*^									0.0214	0.0946 (0.014–0.175)
	Anhui	1	194	30	15.46% (10.69- 20.92)	0.00	–	–		
	Fujian	6	1,905	244	6.25% (0.58- 16.36)	201.70	<0.01	97.5%		
	Guangdong	3	21,641	2,580	15.17% (8.27–23.68)	77.85	<0.01	97.4%		
	Guangxi	1	17	6	35.29% (14.00–59.80)	0.00	–	–		
	Guizhou	5	8,804	403	4.29% (1.10–9.16)	40.06	<0.01	90.0%		
	Henan	1	526	19	3.61% (2.17–5.39)	0.00	–	–		
	Heilongjiang	2	1,107	13	1.11% (0.18- 2.61)	1.39	0.24	28.2%		
	Hubei	6	3,343	361	10.81% (5.14–18.19)	165.32	<0.01	97.0%		
	Hunan	5	2,710	251	7.36% (2.51–14.42)	135.23	<0.01	97.0%		
	Jiangxi	3	3,691	366	7.23% (1.8–15.63)	88.61	<0.01	97.7%		
	Sichuan	4	3,756	241	6.92% (2.59–13.08)	77.59	<0.01	96.1%		
	Yunnan	1	71	7	9.86% (3.84–18.04)	0.00	–	–		
	Zhejiang	8	6,286	1,096	13.70% (9.51–18.51)	128.15	<0.01	94.5%		
Sampling year									0.8036	0.011 (−0.077–0.099)
	1960 to 2009	25	24,908	2,308	8.18% (5.69–11.07)	1,207.41	<0.01	98.0%		
	2010 to 2020	13	5,156	383	7.52% (3.54–12.71)	391.33	<0.01	96.9%		
Detection methods^*^									0.0065	0.124 (0.035–0.213)
	Aetiological testing	31	49,177	5,061	8.24% (6.34–10.34)	1,466.58	<0.01	98.0%		
	Nucleic acid testing	8	1,768	136	9.74% (5.59–14.79)	56.37	<0.01	87.6%		
	Serological testing	7	2,070	433	15.94% (7.94–25.91)	143.73	<0.01	95.8%		
Sex^*^									0.0447	0.058 (0.001–0.115)
	Male	3	2,654	593	21.38% (17.11–26.00)	15.21	<0.01	86.9%		
	Female	3	3,328	574	17.07% (15.34–18.87)	3.59	0.17	44.3%		
Sample^*^									0.0065	0.121 (0.034–0.207)
	Serum	8	2,470	478	15.30% (8.34–23.79)	162.48	<0.01	95.7%		
	Kidney	40	51,813	5,166	7.97% (6.21–9.91)	1,750.52	0	97.8%		
Season									0.4578	0.057 (−0.094–0.209)
	Spring	2	123	7	5.39% (1.82–10.38)	0.82	0.37	0.0%		
	Summer	2	385	32	8.28% (5.69–11.29)	0.02	0.90	0.0%		
	Autumn	7	4,237	340	10.76% (5.45–17.50)	140.80	<0.01	95.7%		
Study quality									0.3729	0.036 (−0.044–0.116)
	Medium	9	28,762	3,615	10.58% (6.95–14.85)	473.25	<0.01	98.3%		
	High	37	25,289	2,002	8.27% (6.25–10.54)	1,119.27	<0.01	96.8%		
Total		46	54,051	5,617	8.70% (6.93–10.64)	0.01	0	98.0%		

* P < 0.05.

CI, confidence interval.

χ^2^ and I^2^, two heterogeneity evaluation indicators, heterogeneity was predicted using I^2^ and Cochrane Q statistics (expressed as χ^2^ and P-values), with an I^2^ value of 25% corresponding to low heterogeneity, 50% to moderate heterogeneity and 75% to high heterogeneity.

**Table 4:** Pooled prevalence of leptospirosis infection by *Leptospira* classification.

**Classification**	**No. studies**	**No. ** ** tested**	**No. positive**	**Prevalence**	**% (95% CI)**	**Heterogeneity**			**Correlation analysis**
						**χ^2^**	***P* value**	***I^2^* (%)**	**R^2^-region**
									47.88%
*Leptospira borgpetersenii* serovar *Ballum*	1	620	1	0.16%	0.00–0.69	0.00	–	–	
*Leptospira kirschneri* serovar *Pomona*	1	1,012	4	0.40%	0.08–0.90	0.00	–	–	
*Leptospira interrogans* serovar *Pyogenes*	1	1,404	1	0.07%	0.00–0.31	0.00	–	–	
*Leptospira interrogans* serovar *Australis*	4	3,746	43	0.92%	0.43–1.56	5.29	0.15	43.3%	
*Leptospira interrogans* serovar *Icterohaemorrhagiae*	16	14,198	1,030	7.05%	4.32–10.37	685.56	<0.01	97.8%	
*Leptospira interrogans* serovar *Grippotyphosa*	4	3,942	9	0.12%	0.00–0.91	19.92	<0.01	84.9%	
*Leptospira interrogans* serovar *Hebdomadis*	2	3,275	2	0.05%	0.00–0.18	0.44	0.51	0.0%	
*Leptospira interrogans* serovar *Autumnalis*	3	3,030	7	0.36%	0.00–1.39	11.30	<0.01	82.3%	
*Leptospira interrogans* serovar *Canicola*	3	3,322	2	0.00%	0.00–0.11	4.63	0.1	56.8%	
*Leptospira interrogans* serovar *Sejroe*	1	333	1	0.30%	0.00–1.29	0.00	–	–	
*Leptospira borgpetersenii* serovar *Javanica*	8	10,606	767	4.73%	2.29–7.97	685.56	<0.01	97.8%	
Total	44	45,488	1,867	8.70%	6.93–10.64	2011.73	0	97.8%	

CI, confidence interval.

χ^2^ and I^2^, two heterogeneity evaluation indicators, heterogeneity was predicted using I^2^ and Cochrane Q statistics (expressed as χ^2^ and P-values), with an I^2^ value of 25% corresponding to low heterogeneity, 50% to moderate heterogeneity and 75% to high heterogeneity.

R^2^, explain the magnitude of heterogeneity.

The Leptospira named according to Adler's reference books and revisions.

**Table 5:** Pooled prevalence of leptospirosis infection in different murine species.

**Classifications**		**No. studies**	**No. tested**	**No. positive**	**% (95% CI)**	**Heterogeneity**			**Correlation analysis**
***genus***	***species***					**χ^2^**	***P*–value**	***I^2^* (%)**	**R^2^-region**
									29.85%
*Apodemus*	*agrarius*	27	29,562	3,646	10.13% (7.39–13.19)	898.17	<0.01	97.1%	
	*peninsulae*	2	111	0	0.00% (0.00–0.00)	0.71	0.40	0.0%	
	**Total**	**29**	**29,673**	**3,646**	**8.35% (5.68–11.37)**	**938.54**	**<0.01**	**97.0%**	
*Bandicota*	*indica*	2	65	15	22.63% (9.95–38.31)	1.88	0.17	46.9%	
*Berylmys*	*bowersi*	5	131	27	12.75% (0.01–36.06)	19.36	<0.01	79.3%	
*Mus*	*musculus Linnaeus*	24	2,803	85	1.82% (0.41–3.87)	115.42	<0.01	80.1%	
*Niviventer*	*coninga*	3	153	4	1.85% (0.04–5.22)	1.23	0.54	0.0%	
	*eha*	2	754	7	0.62% (0.00–1.93)	1.15	0.28	13.2%	
	*fulvescens*	4	330	35	4.76% (5.9–8.8)	7.21	0.07	58.4%	
	*niviventer*	6	534	10	0.00% (0.00–1.58)	7.09	0.21	29.5%	
	**Total**	**15**	**1,771**	**56**	**0.37% (0.00–3.16)**	**67.21**	**<0.01**	**79.2%**	
*Rattus*	*rattus*	2	13	5	29.04% (0.00–76.60)	1.56	0.21	35.9%	
	*flavipectus*	28	2,444	267	5.12% (1.84–9.44)	265.19	<0.01	89.8%	
	*losea*	20	5,792	873	11.95% (8.20–16.20)	226.30	<0.01	91.6%	
	*nitidus*	5	979	37	10.30% (0.00–4.56)	12.20	0.02	67.2%	
	*norvegicus*	35	7,442	467	4.17% (1.81–7.17)	664.84	<0.01	94.9%	
	**Total**	**90**	**16,670**	**1,649**	**5.91% (3.98–8.10)**	**1620.01**	**<0.01**	**94.5%**	

CI, confidence interval.

χ^2^ and I^2^, two heterogeneity evaluation indicators, heterogeneity was predicted using I^2^ and Cochrane Q statistics (expressed as χ^2^ and P-values), with an I^2^ value of 25% corresponding to low heterogeneity, 50% to moderate heterogeneity and 75% to high heterogeneity.

R^2^, explain the magnitude of heterogeneity.

Total, the pooled prevalence of Leptospira in murine gens as a subgroup.

We analyzed the effects of geographic distribution and factors, sampling year, detection method, sex, sample type, season, and study quality on the prevalence of murine leptospirosis (Table 3). All estimates for each subgroup were made using a random-effects model. Among the provinces, the prevalence of murine leptospirosis was the highest in Guangxi Province, at 35.29%, and the lowest in Heilongjiang Province, at 1.11% (Figure 7). In gender subgroups, the prevalence of leptospirosis in males (21.38%, 95%CI: 17.11–26.00%; Table 3) was significantly higher than in females (17.07%, 95%CI: 15.34%−18.87%; *P* < 0.05). Among the three types of assays–etiological, nucleic acid and serological–the serological assay had a higher prevalence (15.94%, 95%CI: 7.94–25.91%, Table 3) and the difference was highly significant (*P* < 0.01). In the sample subgroup, the positive rate of serum samples (15.30%, 95%CI: 8.34–23.79%) was significantly higher than that of kidney samples (7.97%, 95%CI: 6.21–9.91%). Univariate regression analysis indicated that region, detection methods, gender and sample type subgroups were likely major sources of heterogeneity (*P* < 0.05).

We further assessed the prevalence of murine leptospirosis in subgroups of climatic and geographic factors and found that murine leptospirosis ranged from 111–121 degrees longitude (10.45%, 95% CI: 7.91–13.30%), 22–27°C mean temperature (24.44%, 95% CI: 17.93–31.56%) and the basin subgroup (12.72%, 95% CI: 6.82–20.02%) had a higher prevalence, suggesting that these geographic ranges may account for the heterogeneity (Table 6).

**Table 6:** Subgroup analysis of geographical factors.

		**No. studies**	**No. tested**	**No. ** ** positive**	**% (95% CI)**	**Heterogeneity**			**Univariate meta–regression**	
						**χ^2^**	***P*–value**	***I^2^* (%)**	***P*–value**	**Coefficient (95% CI)**
Latitude									0.360	0.049 (−0.055 to 0.153)
	21–25°	7	4276	572	10.91% (6.02–16.94)	139.95	<0.01	95.7%		
	26–30°	25	14,784	1,822	8.68% (6.03–11.75)	822.51	<0.01	97.1%		
	31–35°	6	2,372	184	6.96% (2.96–12.44)	102.17	<0.01	95.1%		
Longitude^*^									0.049	0.088 (0.001 to 0.175)
	100–110°	9	4612	283	5.33% (2.54–8.97)	117.78	<0.01	93.2%		
	111–121°	27	16,820	2,295	10.45% (7.91–13.30)	776.07	<0.01	96.6%		
Altitude									0.307	0.041 (−0.038 to 0.119)
	0–100 m	22	12,901	1,830	10.01% (7.11–13.33)	665.57	<0.01	96.8%		
	101–201 m	15	8,531	754	7.48% (4.70–10.80)	317.21	<0.01	95.6%		
Precipitation									0.336	0.061 (−0.063 to 0.184)
	500–1500 mm	20	11,831	1,301	7.36% (4.57–10.70)	646.47	<0.01	97.1%		
	1501–2500 mm	5	5,173	613	11.14% (5.38–18.62)	211.06	<0.01	98.1%		
Humidity									0.736	0.023 (−0.109 to 0.155)
	60–70%	4	895	57	6.99% (2.13–14.14)	28.61	<0.01	89.5%		
	71–81%	22	16,109	1,857	8.27% (5.62–11.36)	809.72	<0.01	97.42%		
Mean temperature^*^									0.0042	0.279 (0.088 to 0.469)
	10–15	4	1,247	49	2.61% (0.57–5.88)	22.34	<0.01	86.6%		
	16–21	20	15,016	1,685	8.54% (5.9–11.59)	666.97	<0.01	97.2%		
	22–27	2	741	180	24.44% (17.93–31.56)	1.18	0.28	15.6%		
Topography^*^									0.006	−0.1616 (−0.2764 to −0.0468)
	Mountainous	4	1,963	205	10.11% (4.08–18.36)	65.98	<0.01	95.5%		
	Hills	5	1,012	35	2.98% (1.75–4.50)	5.26	0.26	24.0%		
	Plain	7	2,964	244	9.62% (4.55–16.28)	147.75	<0.01	95.9%		
	Basin	6	1,738	231	12.72% (6.82–20.02)	73.65	<0.01	93.2%		

* P < 0.05.

CI, confidence interval.

χ^2^ and I^2^, two heterogeneity evaluation indicators, heterogeneity was predicted using I^2^ and Cochrane Q statistics (expressed as χ^2^ and P values), with an I^2^ value of 25% corresponding to low heterogeneity, 50% to moderate heterogeneity and 75% to high heterogeneity.

## Discussion

Leptospirosis, as a zoonotic manifestation of natural foci disease, is widely distributed in the world, and is most common in tropical and subtropical regions. China has a complex ecological geography and climate, and the natural conditions in most areas are suitable for the survival of pathogenic *Leptospira* and its host animals (3). Therefore, leptospirosis is widely distributed throughout the country, causing great harm to human and animal health. So far, more than 80% of the provinces in China have reported leptospirosis cases, with a total of more than 2.5 million cases reported, including 20,000 deaths (73). Although the overall incidence of leptospirosis had shown a slow downward trend in recent years, there has also been an upward trend in some provinces of China (such as Fujian and Yunnan) (74). Murine have been the main source of infection, and livestock, especially pigs and cattle, and dogs are important hosts and sources of *Leptospira* (3). Therefore, a detailed understanding of the prevalence of murine leptospirosis is of vital importance to prevent human disease and to ensure timely preventative measures are taken.

We analyzed the sampling years first and found that the incidence of leptospirosis had decreased slightly in the past 10 years (Table 3). With the development of the economy and society and attention to and investment in zoonosis, the disease had been controlled nationwide. However, in recent years, with the warming of the global climate, changes in human activities, the variation of pathogens and increased drug resistance in murine (17), reports of the infection in many countries and regions, including some developed countries, have gradually increased, that is, re-emergence of leptospirosis has occurred (75). According to the World Health Organization, leptospirosis, with more than 1 million cases per year, is one of the major zoonotic diseases causing morbidity and mortality worldwide. Therefore, research and prevention of the disease cannot be ignored (14).

Murine are one of the main sources of *Leptospira*, and most of them are recessively infected—they do not get sick themselves, but discharge a large number of pathogens to contaminate the environment and infect humans and other susceptible animals (14, 75–77). Therefore, we analyzed the prevalence of leptospirosis in China and found that it ranged from 1.11 to 35.29%. The combined prevalence of *Leptospira* in murine was 8.7% (Table 4, Figure 7). This showed that the disease has been widespread in China, and there have been large differences across regions. At present, more than 300 serovars of *Leptospira* can be found in the world. The prevalent serovars and distributions differ greatly across regions, and the lack of cross-immunity among them leads to the diagnosis and prevention of the disease having certain regional specificity. Our regional distribution subgroup analysis found that the epidemic area of leptospirosis in China was widely distributed, and the epidemic range covered 5 regions and 14 provinces. However, the prevalence of the infection in murine in southern China was 20.13%, which is significantly higher than in northeast China (*P* < 0.05). Combined with the provincial subgroup analysis, murine leptospirosis was mainly distributed in some provinces in the Yangtze River Basin, and Guangxi Province was particularly affected, with a prevalence rate of 35.29% (Table 4). Guangxi is one of the hardest-hit areas for leptospirosis in China with deaths caused by the disease every year (78). According to reports, epidemics mainly occur in Guangxi rural areas, and the victims are mainly farmers engaged in agricultural labor in the fields for long periods, resulting in repeated exposure to contaminated water (79). In addition, the epidemic forms of leptospirosis in Guangdong and Zhejiang provinces were mainly paddy-field type and rainwater type, and the incidence was mostly in young and middle-aged farmers. Polluted water sources and bacteria-carrying murine are clearly important sources of infection (80). We suggest that murine control, environmental remediation, and publicity of prevention and control knowledge for key occupational groups such as farmers should be periodically carried out during the busy farming season. Popularized the knowledge of leptospirosis transmission routes and susceptible populations, and strengthen the vaccination of high-risk groups. At the same time, strengthen the protection of practitioners, water quality testing should be strengthened to reduce the risk of human infection. We further analyzed the reasons for the low incidence of leptospirosis in Northeast China. All samples from Northeast China collected kidney tissue samples for testing. Combining sample subgroups and detection method subgroups, we found that the results of sample types and detection methods were consistent, and the positive rate of serum samples was higher than that of tissue samples, so the detection method may be one of the reasons for the low incidence in Northeast China. In addition, Northeast China is not the main foci of leptospirosis rodent foci. The natural foci of leptospirosis in the country are mainly distributed in the Yangtze River Basin and the vast areas south of it, which reduces the possibility of disease transmission, which is another reason for its low incidence (75).

The occurrence of murine *Leptospira* was also closely related to climatic and geographical factors, which had important effects on the distribution and population density of host species and reservoirs. These factors determined the species and quantity distribution of host murine, which determined the distribution of pathogens, thereby determining the geographical distribution of the murine *Leptospira* locus. The results of this study showed that the annual average temperature, longitude, and landform characteristics were the main environmental factors affecting the occurrence of murine leptospirosis in China. In the basin area between 111- and 121-degrees east longitude, and temperature 22–27°C, the incidence is highest. These geographical conditions were mainly concentrated in Guangxi, Guangdong, Hubei, Jiangxi, Sichuan and Guizhou. Suitable temperature and altitude, and abundant rainfall determined the distribution of host animals. There are multi-species and great abundance of murine in Guangxi, which are the main source *Leptospira* infection (79). Hubei is the most important rice-growing area in China and, because of frequent floods, the incidence of leptospirosis was relatively high (65). We further analyzed the correlation between *Leptospira* serovars and provincial subgroups, and the R^2^ was 47.88%, indicating that different provinces have a certain influence on *Leptospira* serovars, which may be one of the sources of heterogeneity. Different climatic conditions, soil types and vegetation conditions in the foci, as well as differing host animals, lead to differences in the occurrence and distribution of *Leptospira* in natural foci. Therefore, strengthening the monitoring of environmental factors in and around the foci is an important means of effectively controlling the occurrence and prevalence of the disease.

The composition of murine species in different regions was related to the infection rate with *Leptospira*, and even the same species of animals had very different bacterial serovars and carrier rates in different regions. We analyzed the combined prevalence of leptospirosis infection in different species of murine and found that the combined prevalence of 29.04 and 22.63% in *Rattus rattus* and *Bandicota indica* was the highest, followed by *Berylmys bowersi, Rattus losea, Rattus nitidus and Apodemus agrarius*, the difference was significant (*P* < 0.05). In the classification of different genus, we found that the prevalence of *Bandicota* and *Berylmys* was the highest, and exhibited an extremly distinct difference (*P* < 0.01) (Table 5). The high merged rate for *Rattus rattus* and *Bandicota indica* might be due to the small number of collections, which may have affected the results. Secondly, these two murine species were reported in Guangdong Province, and may be endemic species that are uncommon in other regions. Most of the species in Guangxi Province were *Rattus flavipectus, Apodemus agrarius* and *Rattus norvegicus*. *Rattus flavipectus* is the dominant rat species. The analysis results showed that *Apodemus agrarius, Rattus norvegicus, Rattus losea* and *Rattus flavipectus* were the main host animals of *Leptospira*, and these were widely distributed in various regions (79). We further analyzed the correlation between the murine species and the provincial subgroup, and the R^2^ was 29.85%, indicating that the province had a certain influence on the murine species, which may be one of the sources of its heterogeneity. In addition, the carrier rate was also affected by factors such as gender, and season. Surveys in various places have proved that the male carrier rate is higher than that of females, which might be because the males engage in a wider range of activities, leading to an increased chance of infection. However, in this study, the factors of season did not have a significant impact on the carrier rate. In the seasonal subgroup, the positive rate in summer and autumn was higher than in spring, but the difference between the groups was not significant, which was roughly consistent with the seasonal conclusions of other studies on the incidence of leptospirosis, mainly related to the breeding season and density of rats (Table 6). The size, the chance of mutual contact, the uniformity of sampling distribution and other factors are related, and the monitoring of the murine population should be strengthened during the epidemic season.

We further analyzed the subgroups of *Leptospira* detection methods, and the positive rate for serological detection methods was significantly higher than that of etiological testing methods and molecular biological detection methods (Table 3). Through combined sample type analysis, we found that the sample type was consistent with the results of the detection method, and the positive rate of serum samples was higher than that of tissue samples. At present, the laboratory diagnosis of *Leptospira* still relies on serological methods. Cross agglutination absorption test and microscope agglutination test (MAT) were the two classic test methods used, and it's latter is also the current gold standard for diagnosis (16). MAT was very sensitive in the early stages of infection, which might be one of the reasons for the high seropositivity rate. However, MAT cannot be used for epidemiology and infection monitoring–it has high requirements for operators, as it is necessary to maintain a live culture of strains for a long time during testing, making it difficult to standardize (81). Although the etiological detection method could be used to isolate and cultivate the field isolates, the doubling time of the different *Leptospira* serovars has different logarithmic growth phases, making it impossible to achieve early diagnosis (82). At the same time, the typical invasion characteristics of *Leptospira* or the growth conditions of the respective samples were different, so isolation from different sample types requires different methods, making isolation more difficult (83, 84). Molecular diagnostic techniques such as PCR allow rapid detection of samples with higher sensitivity and specificity. In addition, PCR can quantify the amount or concentration of bacterial DNA present in the sample, which in turn can determine the degree of infection. However, this method must be carried out in the laboratory and requires professional personnel and equipment, thus it is not suitable for field applications (1, 85). In clinical practice, it is recommended that serological methods are used as the main method and a combination of multiple methods for detection, to improve the detection rate and accuracy.

Leptospirosis is listed as III categories of animal epidemic diseases that is harmful to people's health in China (86). The epidemic is the most serious in the southern provinces of China, which is mainly related to its climate, environment, and distribution of host animals. In recent years, reports of leptospirosis have gradually increased. Therefore, disease monitoring of murine should continue, not only to inform specific rodent management strategies, but to give attention also to environmental control and mobilize society as a whole to keep murine densities low. Changes in the living environment of wild animals because of human activities will inevitably lead to increased opportunities for wild animals to be in contact with domestic animals and people. Therefore, increased surveillance of the prevalence of pathogenic *Leptospira* in, especially, small wild mammals, is beneficial to controlling the risk of human leptospirosis outbreaks. It is of great practical significance for the prevention and control of human leptospirosis.

Our study performed a meta-analysis of 46 papers, including 37 (4–5 point) papers and 9 (2–3 point) papers. The paper did not determine the sampling time or random sampling, no factors such as four or more subgroups (Table 2) would affect the score. For further study, we should extract and analyse the specific *Leptospira* serovars that may be carried by the various murine species in more detail, to provide more accurate data, further scientific analysis and a more reliable theoretical basis for future research on leptospirosis.

The advantages of this study lie in the large sample size, large time span, wide regional distribution, clear and definite analysis method and comprehensive risk factor analysis, which allow for some feasible prevention and control suggestions to be made for the leptospirosis epidemic. However, our meta-analysis had some limitations that may have affected the results. First, although a search formula to comprehensively collect relevant studies had been developed, omissions may have occurred. Second, a large number of eligible studies were obtained in our systematic study, but not all data were available, and insufficient research on murine *Leptospira* subgroups such as age and gender would have affected the results. Thirdly, a limited number (*n* = 2) of the qualified studies were conducted in northeast China, which may not reflect the actual positivity rate in the areas investigated; the quality of those studies was mixed, suggesting that better surveillance and more research on *Leptospira* infection in those areas is required. Fourth, the data available for analysis were limited, particularly for living environment, the existence of mixed infection, and the predominant murine and prevalent strains in each region, and further, more detailed analysis has not been carried out. Despite these limitations, we believe this report is an accurate reflection of murine *Leptospira* infection prevalence in China.

## Conclusion

*Leptospira* was found to be widespread in China. Region, sample type, testing method, gender and geographic factors influenced the prevalence of leptospirosis. Formulating a prevention and control strategy for murine leptospirosis that accounts for the differences in climate and environment across regions is suggested. At the same time, epidemiological surveys of *Leptospira* in murine are needed in more areas to further explore the risk factors and to prevent the spread of the disease in humans and animals.

## Data availability statement

The original contributions presented in the study are included in the article/Supplementary material, further inquiries can be directed to the corresponding authors.

## Author contributions

RD: idea contributions. L-ML, J-FS, TL, Q-XM, and WZ: data extraction. H-FF: database establishment. QW: data analysis. J-ML: writing original draft. FL: writing—review and editing. All authors contributed to manuscript editing and approved the final manuscript.

## Conflict of interest

The authors declare that the research was conducted in the absence of any commercial or financial relationships that could be construed as a potential conflict of interest.

## Publisher's note

All claims expressed in this article are solely those of the authors and do not necessarily represent those of their affiliated organizations, or those of the publisher, the editors and the reviewers. Any product that may be evaluated in this article, or claim that may be made by its manufacturer, is not guaranteed or endorsed by the publisher.
