# Supplementary material for: Prevalence of Leptospira in murine in China: A systematic review and meta-analysis
Source: Front Vet Sci. 2022 Sep 29;9:944282. doi: 10.3389/fvets.2022.944282 (PMC9557099; doi:10.3389/fvets.2022.944282)
Supplement: Supplementary file 1 [file Data_Sheet_1.docx]

**Table S1. PRISMA Checklist item.**

| **Section/topic** | **#** | **Checklist item** | **Reported on page #** |
| --- | --- | --- | --- |
| **TITLE** | | |  |
| Title | 1 | Prevalence of Leptospira in murine in China: A systematic review and meta-analysis | 1 |
| **ABSTRACT** | | |  |
| Structured summary | 2 | Provide a structured summary including, as applicable: background; objectives; data sources; study eligibility criteria, participants, and interventions; study appraisal and synthesis methods; results; limitations; conclusions and implications of key findings; systematic review registration number. | 2 |
| **INTRODUCTION** | | |  |
| Rationale | 3 | Describe the rationale for the review in the context of what is already known. | 4 |
| Objectives | 4 | Provide an explicit statement of questions being addressed with reference to participants, interventions, comparisons, outcomes, and study design (PICOS). | 5 |
| **METHODS** | | |  |
| Eligibility criteria | 6 | Specify study characteristics (e.g., PICOS, length of follow-up) and report characteristics (e.g., years considered, language, publication status) used as criteria for eligibility, giving rationale. | 6 |
| Information sources | 7 | Describe all information sources (e.g., databases with dates of coverage, contact with study authors to identify additional studies) in the search and date last searched. | 6 |
| Search | 8 | Present full electronic search strategy for at least one database, including any limits used, such that it could be repeated. | 6, Table S2 |
| Study selection | 9 | State the process for selecting studies (i.e., screening, eligibility, included in systematic review, and, if applicable, included in the meta-analysis). | 6, Figure 1 |
| Data collection process | 10 | Describe method of data extraction from reports (e.g., piloted forms, independently, in duplicate) and any processes for obtaining and confirming data from investigators. | 6, Additional file. |
| Data items | 11 | List and define all variables for which data were sought (e.g., PICOS, funding sources) and any assumptions and simplifications made. | 6, Additional file. |
| Risk of bias in individual studies | 12 | Describe methods used for assessing risk of bias of individual studies (including specification of whether this was done at the study or outcome level), and how this information is to be used in any data synthesis. | 7 |
| Summary measures | 13 | State the principal summary measures (e.g., risk ratio, difference in means). | 7 |
| Synthesis of results | 14 | Describe the methods of handling data and combining results of studies, if done, including measures of consistency (e.g., I^2^) for each meta-analysis. | 7 |

Page 1 of 2

| **Section/topic** | **#** | **Checklist item** | **Reported on page #** |
| --- | --- | --- | --- |
| Risk of bias across studies | 15 | Specify any assessment of risk of bias that may affect the cumulative evidence (e.g., publication bias, selective reporting within studies). | 7 |
| Additional analyses | 16 | Describe methods of additional analyses (e.g., sensitivity or subgroup analyses, meta-regression), if done, indicating which were pre-specified. | 7，Figure 6 |
| **RESULTS** | | |  |
| Study selection | 17 | Give numbers of studies screened, assessed for eligibility, and included in the review, with reasons for exclusions at each stage, ideally with a flow diagram. | 8, Figure 1 |
| Study characteristics | 18 | For each study, present characteristics for which data were extracted (e.g., study size, PICOS, follow-up period) and provide the citations. | 8, Additional file. |
| Risk of bias within studies | 19 | Present data on risk of bias of each study and, if available, any outcome level assessment (see item 12). | 8, Additional file. |
| Results of individual studies | 20 | For all outcomes considered (benefits or harms), present, for each study: (a) simple summary data for each intervention group (b) effect estimates and confidence intervals, ideally with a forest plot. | 9, Additional file. |
| Synthesis of results | 21 | Present results of each meta-analysis done, including confidence intervals and measures of consistency. | 8-9, Table 2-3 |
| Risk of bias across studies | 22 | Present results of any assessment of risk of bias across studies (see Item 15). | 9-10, Figure 2. |
| Additional analysis | 23 | Give results of additional analyses, if done (e.g., sensitivity or subgroup analyses, meta-regression [see Item 16]). | 10, Figure S3-s11. |
| **DISCUSSION** | | |  |
| Summary of evidence | 24 | Summarize the main findings including the strength of evidence for each main outcome; consider their relevance to key groups (e.g., healthcare providers, users, and policy makers). | 10-16 |
| Limitations | 25 | Discuss limitations at study and outcome level (e.g., risk of bias), and at review-level (e.g., incomplete retrieval of identified research, reporting bias). | 16 |
| Conclusions | 26 | Provide a general interpretation of the results in the context of other evidence, and implications for future research. | 17 |
| **FUNDING** | | |  |
| Funding | 27 | Describe sources of funding for the systematic review and other support (e.g., supply of data); role of founders for the systematic review. | 18 |

*From:*  Moher D, Liberati A, Tetzlaff J, Altman DG, The PRISMA Group (2009). Preferred Reporting Items for Systematic Reviews and Meta-Analyses: The PRISMA Statement. PLoS Med 6(6): e1000097. doi:10.1371/journal.pmed1000097

For more information, visit: **www.prisma-statement.org**.

Page 2 of 2

**Table S2.** **Search strategy.**

| Database | Search Strategy |
| --- | --- |
| PubMed database | In the PubMed database, the Boolean operators "AND" and "OR" were commonly used to connect the theme words and the free words, respectively. According to terms deposited in the MeSH database, the following keywords were used to search for the articles: “Mice”, “*Leptospira*” and “China”.  We used three retrieval formulas. Formula A was as follows: (((((((((((((((((("Mice"[Mesh]) OR (Mus)) OR (Mouse)) OR (Mus domesticus)) OR (Mus musculus domesticus)) OR (domesticus, Mus musculus)) OR (Mus musculus)) OR (Mice, House)) OR (House Mice)) OR (Mouse, House)) OR (House Mouse)) OR (Mouse, Swiss)) OR (Swiss Mouse)) OR (Swiss Mice)) OR (Mice, Swiss)) OR (Mice, Laboratory)) OR (Laboratory Mice)) OR (Mouse, Laboratory)) OR (Laboratory Mouse). Retrieval Formula B was as follows: ((((“China” [Mesh]) OR People's Republic of China) OR Mainland China) OR Manchuria) OR Sinkiang) OR Inner Mongolia. Retrieval formula C was: ("*Leptospira*"[Mesh]).  We connected the formulas A, B and C with the Boolean operator "AND", and the final search formula was ("*Leptospira*"[Mesh]) AND ((((((((((((((((((("Mice"[Mesh]) OR (Mus)) OR (Mouse)) OR (Mus domesticus)) OR (Mus musculus domesticus)) OR (domesticus, Mus musculus)) OR (Mus musculus)) OR (Mice, House)) OR (House Mice)) OR (Mouse, House)) OR (House Mouse)) OR (Mouse, Swiss)) OR (Swiss Mouse)) OR (Swiss Mice)) OR (Mice, Swiss)) OR (Mice, Laboratory)) OR (Laboratory Mice)) OR (Mouse, Laboratory)) OR (Laboratory Mouse)) AND (((((“China” [Mesh]) OR People's Republic of China) OR Mainland China) OR Manchuria) OR Sinkiang) OR Inner Mongolia. |
| ScienceDirect | In the ScienceDirect database, the keywords “China”, “Mice”, “*Leptospira*” “epidemiology” and “prevalence” were used to search. |
| CNKI; VIP; Wanfang | In the Chinese databases, the search terms “*Leptospira*” (in Chinese) and “Mice” (in Chinese) were used for advanced search. We used fuzzy search and synonym expansion for all the databases. |

**Table S3. Egger’s for publication bias**

| t | p-value | bias | se.bias | slope |
| --- | --- | --- | --- | --- |
| -0.489 | 0.628 | -0.697 | 1.428 | 0.324 |

**Table S4.** Pooled prevalence of leptospirosis infection in different murine species.

| Classifications | | No.  studies | No.  tested | No.  positive | % (95% CI) | Heterogeneity | | | Univariate meta-regression of  murine genus | | Univariate meta-regression of  murine species | | Correlation Analysis |
| --- | --- | --- | --- | --- | --- | --- | --- | --- | --- | --- | --- | --- | --- |
| *genus* | *species* |  |  |  |  | χ² | *P* value | *I²* (%) | *P* value | % (95% CI) | *P* value | % (95% CI) | R^2^-region |
|  |  |  |  |  |  |  |  |  | 0.0008 | -0.1125 (-0.1782- -0.0469) | 0.0445 | 0.347 (0.009- 0.686) | 29.85% |
| *Apodemus* | *agrarius* | 27 | 29,562 | 3,646 | 10.13% (7.39-13.19) | 898.17 | <0.01 | 97.1% |  |  |  |  |  |
|  | *peninsulae* | 2 | 111 | 0 | 0.00% (0.00-0.00) | 0.71 | 0.40 | 0.0% |  |  |  |  |  |
|  | **Total** | **29** | **29,673** | **3,646** | **8.35% (5.68-11.37)** | **938.54** | **<0.01** | **97.0%** |  |  |  |  |  |
| *Bandicota* | *indica* | 2 | 65 | 15 | 22.63% (9.95-38.31) | 1.88 | 0.17 | 46.9% |  |  |  |  |  |
| *Berylmys* | *bowersi* | 5 | 131 | 27 | 12.75% (0.01-36.06) | 19.36 | <0.01 | 79.3% |  |  |  |  |  |
| *Mus* | *musculus* *Linnaeus* | 24 | 2,803 | 85 | 1.82% (0.41-3.87) | 115.42 | <0.01 | 80.1% |  |  |  |  |  |
| *Niviventer* | *coninga* | 3 | 153 | 4 | 1.85% (0.04-5.22) | 1.23 | 0.54 | 0.0% |  |  |  |  |  |
|  | *eha* | 2 | 754 | 7 | 0.62% (0.00-1.93) | 1.15 | 0.28 | 13.2% |  |  |  |  |  |
|  | *fulvescens* | 4 | 330 | 35 | 4.76% (5.9-8.8) | 7.21 | 0.07 | 58.4% |  |  |  |  |  |
|  | *niviventer* | 6 | 534 | 10 | 0.00% (0.00-1.58) | 7.09 | 0.21 | 29.5% |  |  |  |  |  |
|  | **Total** | **15** | **1,771** | **56** | **0.37% (0.00-3.16)** | **67.21** | **<0.01** | **79.2%** |  |  |  |  |  |
| *Rattus* | *rattus* | 2 | 13 | 5 | 29.04% (0.00-76.60) | 1.56 | 0.21 | 35.9% |  |  |  |  |  |
|  | *flavipectus* | 28 | 2,444 | 267 | 5.12% (1.84-9.44) | 265.19 | <0.01 | 89.8% |  |  |  |  |  |
|  | *losea* | 20 | 5,792 | 873 | 11.95% (8.20-16.20) | 226.30 | <0.01 | 91.6% |  |  |  |  |  |
|  | *nitidus* | 5 | 979 | 37 | 10.30% (0.00-4.56) | 12.20 | 0.02 | 67.2% |  |  |  |  |  |
|  | *norvegicus* | 35 | 7,442 | 467 | 4.17% (1.81-7.17) | 664.84 | <0.01 | 94.9% |  |  |  |  |  |
|  | **Total** | **90** | **16,670** | **1,649** | **5.91% (3.98-8.10)** | **1620.01** | **<0.01** | **94.5%** |  |  |  |  |  |

CI: confidence interval.

χ² and *I*^2^: two heterogeneity evaluation indicators, heterogeneity was predicted using *I^2^* and Cochrane Q statistics (expressed as χ² and *P* values), with an *I^2^* value of 25% corresponding to low heterogeneity, 50% to moderate heterogeneity and 75% to high heterogeneity.

R^2^: explain the magnitude of heterogeneity.

Total: the pooled prevalence of *Leptospira* in murine gens as a subgroup.

**Table S5.** Pooled prevalence of leptospirosis infection by *Leptospira* classification.

| Classification | No.  studies | No.  tested | No.  positive | Prevalence | % (95% CI) | Heterogeneity | | | Univariate meta-regression | | Correlation Analysis |
| --- | --- | --- | --- | --- | --- | --- | --- | --- | --- | --- | --- |
|  |  |  |  |  |  | χ² | *P* value | *I²* (%) | *P* value | % (95% CI) | R^2^-region |
|  |  |  |  |  |  |  |  |  | 0.1180 | -0.148 (-0.330-0.034) | 47.88% |
| *Leptospira borgpetersenii serovar Ballum* | 1 | 620 | 1 | 0.16% | 0.00-0.69 | 0.00 | -- | -- |  |  |  |
| *Leptospira kirschneri serovar Pomona* | 1 | 1,012 | 4 | 0.40% | 0.08-0.90 | 0.00 | -- | -- |  |  |  |
| *Leptospira interrogans serovar Pyogenes* | 1 | 1,404 | 1 | 0.07% | 0.00-0.31 | 0.00 | -- | -- |  |  |  |
| *Leptospira interrogans serovar* *Australis* | 4 | 3,746 | 43 | 0.92% | 0.43-1.56 | 5.29 | 0.15 | 43.3% |  |  |  |
| *Leptospira interrogans serovar Icterohaemorrhagiae* | 16 | 14,198 | 1,030 | 7.05% | 4.32-10.37 | 685.56 | <0.01 | 97.8% |  |  |  |
| *Leptospira interrogans serovar Grippotyphosa* | 4 | 3,942 | 9 | 0.12% | 0.00-0.91 | 19.92 | <0.01 | 84.9% |  |  |  |
| *Leptospira interrogans serovar Hebdomadis* | 2 | 3,275 | 2 | 0.05% | 0.00-0.18 | 0.44 | 0.51 | 0.0% |  |  |  |
| *Leptospira interrogans serovar Autumnalis* | 3 | 3,030 | 7 | 0.36% | 0.00-1.39 | 11.30 | <0.01 | 82.3% |  |  |  |
| *Leptospira interrogans serovar Canicola* | 3 | 3,322 | 2 | 0.00% | 0.00-0.11 | 4.63 | 0.1 | 56.8% |  |  |  |
| *Leptospira interrogans serovar Sejroe* | 1 | 333 | 1 | 0.30% | 0.00-1.29 | 0.00 | -- | -- |  |  |  |
| *Leptospira borgpetersenii serovar Javanica* | 8 | 10,606 | 767 | 4.73% | 2.29-7.97 | 685.56 | <0.01 | 97.8% |  |  |  |
| Total | 44 | 45,488 | 1,867 | 8.70% | 6.93-10.64 | 2011.73 | 0 | 97.8% |  |  |  |

CI: confidence interval.

χ² and *I*^2^: two heterogeneity evaluation indicators, heterogeneity was predicted using *I^2^* and Cochrane Q statistics (expressed as χ² and *P* values), with an *I^2^* value of 25% corresponding to low heterogeneity, 50% to moderate heterogeneity and 75% to high heterogeneity.

R^2^: explain the magnitude of heterogeneity.

The *Leptospira* named according to Adler´s reference books and revisions.

**Figure S1. Funnel plot with pseudo 95% confidence limits intervals for the examination of publication bias of regions**

**Figure S2. Funnel plot with pseudo 95% confidence limits intervals for the examination of publication bias of sampling years**

**Figure S3. Funnel plot with pseudo 95% confidence limits intervals for the examination of publication bias of province**

**Figure S4. Funnel plot with pseudo 95% confidence limits intervals for the examination of publication bias of *Leptospira* typing**

**Figure S5. Funnel plot with pseudo 95% confidence limits intervals for the examination of publication bias of gender**

**Figure S6. Funnel plot with pseudo 95% confidence limits intervals for the examination of publication bias of sample classification**

**Figure S7. Funnel plot with pseudo 95% confidence limits intervals for the examination of publication bias of detection method**

**Figure S8. Funnel plot with pseudo 95% confidence limits intervals for the examination of publication bias of season**

**Figure S9. Funnel plot with pseudo 95% confidence limits intervals for the examination of publication bias of murine species**

**Figure S10. Funnel plot with pseudo 95% confidence limits intervals for the examination of publication bias of annual average temperature**

**Figure S11. Funnel plot with pseudo 95% confidence limits intervals for the examination of publication bias of study quality**
